# First Insights on the Presence of the Unfolded Protein Response in Human Spermatozoa

**DOI:** 10.3390/ijms20215518

**Published:** 2019-11-05

**Authors:** Joana Santiago, Joana Vieira Silva, Margarida Fardilha

**Affiliations:** 1 Laboratory of Signal Transduction, Department of Medical Sciences, Institute of Biomedicine—iBiMED, University of Aveiro, 3810-193 Aveiro, Portugal; joanasantiago@ua.pt (J.S.); joanavieirasilva@ua.pt (J.V.S.); 2 Reproductive Genetics and Embryo-fetal Development Group, Institute for Innovation and Health Research (I3S), University of Porto, 4200-135 Porto, Portugal; 3 Laboratory of Cell Biology, Department of Microscopy, and Unit for Multidisciplinary Research in Biomedicine (UMIB), Institute of Biomedical Sciences Abel Salazar (ICBAS), University of Porto, 4050-313 Porto, Portugal

**Keywords:** response to unfolded proteins, spermatozoa, oxidative stress

## Abstract

The unfolded protein response (UPR) is involved in protein quality control and is activated in response to several stressors. Although in testis the UPR mechanisms are well described, their presence in spermatozoa is contentious. We aimed to investigate the presence of UPR-related proteins in human sperm and the impact of oxidative stress induction in UPR activation. To identify UPR-related proteins in human sperm, a bioinformatic approach was adopted. To explore the activation of UPR, sperm were exposed to hydrogen peroxide (H_2_O_2_) and motility, vitality, and the levels of UPR-related proteins were assessed. We identified 97 UPR-related proteins in human sperm and showed, for the first time, the presence of HSF1, GADD34, and phosphorylated eIF2α. Additionally, the exposure of human sperm to H_2_O_2_ resulted in a significant decrease in sperm viability and motility and an increase in the levels of HSF1, HSP90, HSP60, HSP27, and eIF2α; all proteins involved in sensing and response to unfolded proteins. This study gave us a first insight into the presence of UPR mechanisms in the male gamete. However, the belief that sperm are devoid of transcription and translation highlight the need to clarify if these pathways are activated in sperm in the same way as in somatic cells.

## 1. Introduction

In cells, protein homeostasis maintenance is crucial. The dysregulation of these mechanisms often impacts the proper biogenesis, folding, traffic, and degradation of proteins in cells [1]. The constant exposure of the cytosol, endoplasmic reticulum (ER), and mitochondria to nascent polypeptides requires dedicated protein-folding machinery that determines an organelle’s protein-folding capacity [1]. When the amount of unfolded or misfolded proteins exceeds the protein-folding capacity, specific signaling pathways are activated in cells, commonly known as the unfolded protein response (UPR). The UPR is a conserved and essential cellular pathway involved in protein quality control and is activated in response to several cellular stressors such as diseases, aging, heat, and toxins [2,3]. Under stress conditions, one of the three lines of defense can be activated: (i) Cytosolic heat shock response (HSR); (ii) endoplasmic reticulum UPR (UPR^ER^); and/or (iii) mitochondrial UPR (UPR^mt^), often requiring the communication with the nuclei [4]. The response to unfolded or misfolded proteins generally culminates in the transcription of heat shock genes (*HSP90A, HSPA, HSPD1,* and small heat shock protein *HSP27*), molecular chaperones that assist the proper folding of proteins [4,5]. Additionally, mechanisms that result in the blockage of protein synthesis, through the phosphorylation of eukaryotic translation initiation factor-2 (eIF2α), to avoid the accumulation of misfolded proteins, are also triggered [6,7].

The male gametes—spermatozoa—are a unique type of cell characterized for its small size, lack of most organelles, high polarity, and fast motility [8]. This type of cell is produced in testis and results from spermatogenesis, a highly regulated process that takes place within the seminiferous tubules [9]. In testis, there is a high rate of protein synthesis and the UPR mechanisms responsible for detecting un- or misfolded proteins, resulting from the exposure of heat, drugs, environmental chemicals, and other sources, are well described [10,11,12]. However, the presence of proteins involved in UPR in spermatozoa is contentious, especially since they are virtually transcriptionally inactive and their translational machinery seems to be lost with the residual cytoplasm, during spermatogenesis. In fact, since sperm cells are deprived of ER, leaving the spermatozoa uncapable of doing protein synthesis, the UPR will probably be more prominent in the cytosol and mitochondria. However, whether these pathways are active in ejaculated spermatozoa is still not clear. 

In the present work, we aimed to investigate the presence of UPR components in human spermatozoa and evaluate the activation of these stress response pathways in response to oxidative stress in human ejaculated spermatozoa. We reported that sperm cells have several UPR-related proteins and showed, for the first time, the presence of heat shock factor 1 (HSF1) and DNA damage-inducible protein (GADD34) proteins in ejaculated human spermatozoa. Additionally, we described that the levels of several UPR-related proteins increased after oxidative stress induction, similarly to what happens in somatic cells, suggesting the activation of the UPR pathway in human spermatozoa.

## 2. Results

### 2.1. UPR-Related Proteins are Present in Ejaculated Human Spermatozoa

To generate a list of human sperm proteins, we collected the outcomes of all human ejaculated sperm proteomic studies and selected the proteins that accomplished the criteria mentioned. Ten independent studies and two reviews, comprising several proteomic studies, were included in this study (Table 1), including both whole cell analysis and subcellular fractioning. By merging data from the available papers, and after removal of duplicates, a list of 7622 different proteins, along with their literature references, was created, that we believe are very likely to exist in human spermatozoa (Table S1: Human sperm proteome).

**Figure 1:**
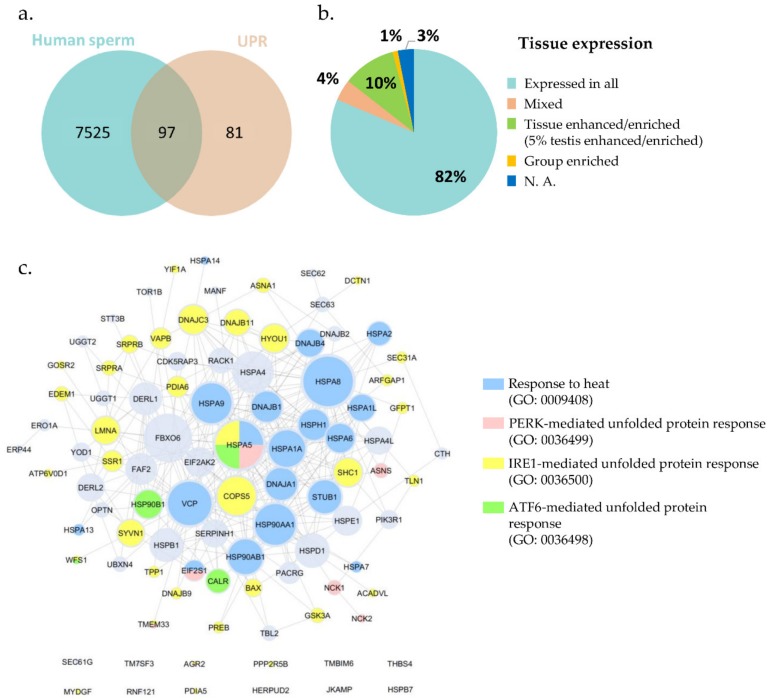
Proteins involved in the response to unfolded protein present in human sperm. (**a**) The compilation of human sperm proteomes resulted in a list of 7622 different proteins. Crossing the sperm proteome with the UPR protein list, from the 178 proteins annotated in the UPR, 97 were present in the ejaculated human sperm. (**b**) Tissue expression distribution of the 97 UPR-related proteins. The 97 UPR proteins mapped in human sperm were annotated in the Human Protein Atlas (HPA) repository. (**c**) Network of the UPR-related proteins already identified in human spermatozoa. Proteins were annotated according to their association with specific GO terms associated with response to unfolded proteins; PERK/IRE1/ATF6-mediated UPR are child terms of UPR^ER^ (GO: 0030968); there were no human proteins annotated in the mitochondrial UPR (GO: 0034515).

The entry GO:0006986, corresponding to the term “response to unfolded protein”, initially retrieved 302 annotations, restricted to Homo sapiens. After excluding all duplicates, a list containing 178 proteins linked to the response to unfolded protein was created (Table S2: UPR-related proteins). Gathering the human sperm proteome and crossing it with the UPR protein list, from the 178 proteins annotated as involved in UPR, 97 were present in human sperm (Figure 1, Table S3: Sperm UPR-related proteins). 

From the 97 UPR proteins mapped in human sperm, 82% were annotated in the Human Protein Atlas (HPA) repository, as expressed in all tissues; 10% were tissue enriched or enhanced (5% testis enriched/enhanced); 4% had mixed expression; and around 1% were group enriched (Figure 1b, Table S3: Sperm UPR-related proteins). Regarding subcellular location, most of those proteins were localized in the cytosol (34%), nucleoplasm (21%), and ER (20%) (Table S3: Sperm UPR-related proteins). Since sperm cells lost the ER during spermiogenesis, the presence of proteins such as DERL1, DERL2, and HSP90B1 in mature spermatozoa should be explored and their localizations investigated. It is plausible to hypothesize that these proteins occupy a different subcellular location and also play different roles in human spermatozoa. 

According to the Online Mendelian Inheritance in Man (OMIM), a database that contains the collection of human genes and genetic disorders and traits associated, only 24 out of the 97 proteins identified were associated to a disease. However, none of them presented an infertility-associated phenotype (Table S3: Sperm UPR-related proteins). *BAX*, *LMNA*, and *WFS1* were associated with an infertility-related condition in DisGeNET database. Nevertheless, 10 proteins (BAX, DERL2, DNAJA1, HSP90AA1, HSPA2, HSPA4, HSPA4L, HSPA5, LMNA, and STUB1) were also associated to infertility-related phenotypes in KO mouse models, comprising asthenozoospermia, azoospermia, arrest of spermatogenesis, abnormal spermatogenesis, abnormal Sertoli cells morphology, and others (Figure S3: Sperm UPR-related proteins). 

The network analysis (Figure 1c) showed that HSPA5 (BiP), HSPA8, and FBXO6 were the proteins with the larger number of interactions, as well as several heat shock proteins (HSPs) such as HSPA4, HSP90AA1, HSP90AB1, HSPD1 (HSP60), HSPE1(HSP10), and others. It is possible to observe that from the 97 UPR-related proteins identified in human sperm, 20 were annotated as involved in “response to heat” (GO: 0009498; blue), 35 involved in “IRE1-mediated unfolded protein response” (GO: 0036500; yellow), 7 involved in “PERK-mediated unfolded protein response” (GO: 0036499; pink), and only 4 annotated in “ATF6-mediated unfolded protein response” (GO: 0036498; green). However, there were no human proteins annotated in the mitochondrial UPR (GO: 0034515) (Table S4: UPR-specific processes).

### 2.2. Identification of HSF1, GADD34, and Phosphorylated eIF2α in Human Spermatozoa for the First Time

**Figure 2:**
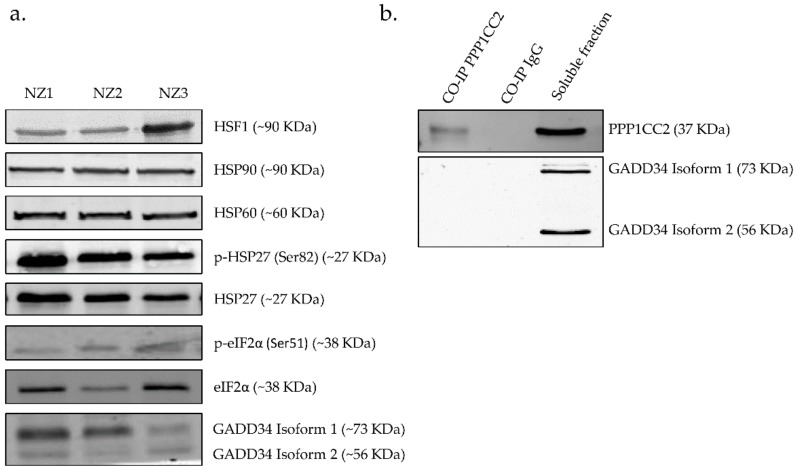
(**a**) Identification of proteins involved in response to unfolded proteins in ejaculated human sperm, and the identification of HSF1, p-eIF2α, and GADD34 in ejaculated human spermatozoa of three normozoospermic (NZ) men for the first time. A total of 30 µg of proteins from whole cell lysates were loaded and separated by SDS-PAGE. (**b**) Co-immunoprecipitation of PPP1CC2 and immunodetection of GADD34 in human ejaculated spermatozoa. PPP1CC2 and GADD34 do not interact in human spermatozoa. Image representative of three independent experiments.

Antibodies against proteins that participate in the response to unfolded proteins (HSP90, HSP60, p-HSP27 (Ser 82), HSP27, HSF1, GADD34, p-eIF2α (Ser 51), eIF2α) were used. To accurately evaluate the results, human testis was used as a positive control since the presence of these proteins has been previously described. All antibodies detected the proteins corresponding to the molecular weight expected. Here we showed that sperm samples contain proteins involved in the response to stress and we identified, for the first time, the presence of HSF1 and GADD34 proteins, as well as the phosphorylated state of eIF2α in ejaculated human spermatozoa (Figure 2a). 

Most of the signaling mechanisms here explored culminate in the transcription of heat shock genes or in the blockage of protein translation, through the phosphorylation of eIF2α at Ser51. On the other hand, the complex GADD34–serine/threonine-protein phosphatase 1 (PPP1) is responsible for restoring the synthesis of new proteins [25]. Indeed, GADD34 recruits PPP1 to dephosphorylate eIF2α, reversing the shut-off of protein synthesis and facilitating the recovery of cells from stress. We investigate the possible interaction between GADD34 and PPP1CC2, a testis-enriched and sperm-specific isoform of PPP1 highly abundant in human spermatozoa [26,27]. However, despite the existence of both proteins in ejaculated human spermatozoa, we failed to detect the interaction between those proteins using a co-immunoprecipitation approach (Figure 2b).

### 2.3. H_2_O_2_ Affects Human Sperm Viability and Motility

**Figure 3:**
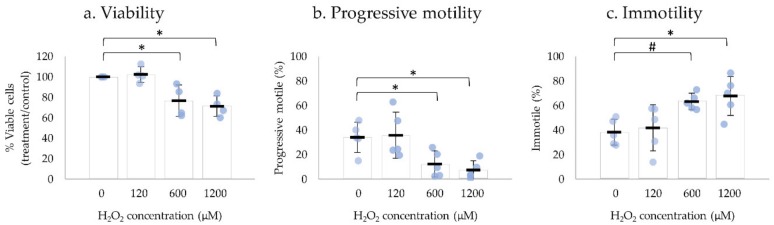
Impact of H_2_O_2_ on human sperm viability and motility. Sperm cells were incubated with 120, 600, and 1200 µM of H_2_O_2_ for 2 h, 15 min, and 30–60 sec, respectively. The viable cells of the control sample (0 µM) was considered 1. Results are expressed as mean ± SD values from four independent experiments for viability and five independent experiments for motility. Statistically significant differences from the relative control (0 µM) are indicated (* *p* < 0.05; # *p* < 0.01).

H_2_O_2_ is one of the most damaging reactive oxygen species (ROS) to human spermatozoa, and its impact on sperm motility has been extensively reported [28,29]. Human ejaculated spermatozoa were exposed to different concentrations of H_2_O_2_ (120, 600, and 1200 µM) and motility parameters and viability were assessed after 2 h, 15 min, and 30–60 sec, respectively (Figure 3). Negative control was performed in the absence of H_2_O_2_.

The incubation with 120 µM of H_2_O_2_ had no significant effect on sperm motility or on the percentage of viable spermatozoa after 2 h incubation. The exposure of human ejaculated spermatozoa to 600 µM H_2_O_2_ for 15 min and 1200 µM of H_2_O_2_ immediately resulted in a significant decrease of viability and progressive motile spermatozoa, thus an increase in immotile spermatozoa compared with the negative control. No significant alterations were observed in non-progressive motility (Table S5: Impact of H_2_O_2_ on sperm motility, viability, and UPR-related protein levels). 

### 2.4. UPR-Related Proteins’ Levels Significantly Increased after Exposure to H_2_O_2_

**Figure 4:**
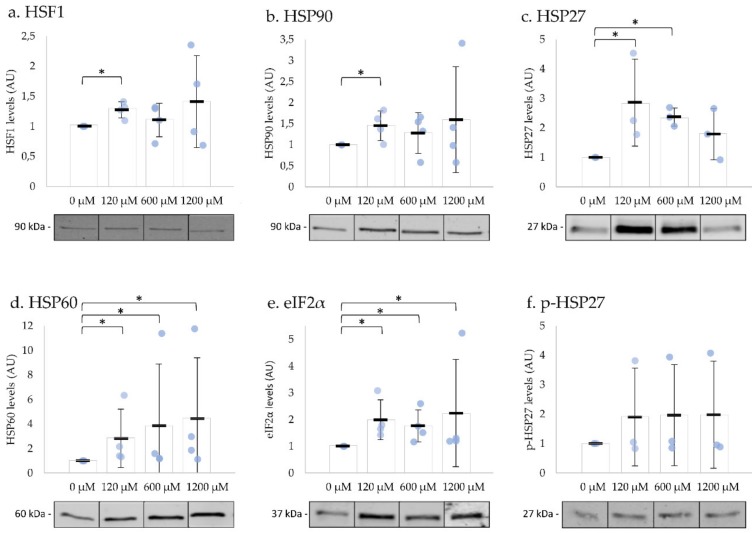
Impact of H_2_O_2_ on human spermatozoa UPR-related proteins’ levels. The levels of HSF1 (**a**), HSP90 (**b**), and HSP27 (**c**), proteins involved in HSR, significantly increased after incubation with low concentrations of H_2_O_2_. HSP60 (**d**) and the total amount of eIF2α (**e**) significantly increased after exposure to all the concentrations tested. The levels of phosphorylated HSP27 (**f**) did not change with any concentration tested. Results are expressed as mean ± SD values from three or four independent experiments. Statistically significant differences from the relative control (0 µM) are indicated (* *p* < 0.05).

To investigate whether UPR is activated in ejaculated human spermatozoa, we incubated the cells with different concentrations of H_2_O_2_ at 37 °C. After exposure to 120 µM H_2_O_2_ for 2 h, the levels of HSF1, HSP90, HSP60, HSP27, and eIF2α significantly increased (Figure 4a–e). The exposure to 600 µM H_2_O_2_ for 15 min resulted in a significant increase of HSP27, HSP60, and eIF2α in comparison to the negative control (Figure 4c–e). The incubation with 1200 µM H_2_O_2_ immediately resulted in an increase in HSP60 and eIF2α levels, both involved in UPR^mt^ (Figure 4d,e). The levels of phosphorylated HSP27 did not change with any concentration tested (Figure 4f; Table S5: Impact of H_2_O_2_ on sperm motility, viability, and UPR-related protein levels). The levels of phosphorylated eIF2α were below detection limits. These results suggested that the UPR were activated after oxidative stress induction in human spermatozoa.

## 3. Discussion

The UPR is a conserved mechanism that comprises the cytosolic HSR [30], the UPR^ER^ [3,5], and the UPR^mt^ [31,32]. These signaling pathways act in response to aberrant protein folding and are controlled by multiple proteins, some of them specific from each pathway. However, all the three responses are mediated by HSP, the sensors of unfolded proteins responsible for the signal activation and that help in the proper folding of proteins [33]. In the last years, the research of UPR in the male reproductive system has been mainly focused on the testis. The activation of HSR and UPR^ER^ under hyperthermia conditions [10,33,34], as well as the response after exposure to chemicals, were showed in several studies in mice testis [11,35]. The belief that spermatozoa are virtually devoid of transcription and translation, as well as the observation that transcriptional machinery is lost during spermatogenesis [36], points to the need for an in-depth study of UPR proteins present in spermatozoa and the need to clarify if the stress response pathways activated in response to unfolded proteins in somatic cells are also activated in spermatozoa.

The collection and mapping of the UPR key elements in human spermatozoa is crucial to establish whether these proteins are present or not. The identification of 97 UPR-related proteins in human spermatozoa gave us a first insight about the presence of these mechanisms in spermatozoa. However, the presence of these proteins, that mainly include molecular chaperones and transcription factors, can result from the spermatogenic process that occur in testis and may not be functionally active in mature spermatozoa. The analysis of UPR proteins’ localization revealed very interesting results, since sperm cells lose most of the cytoplasm and organelles, including ER, during spermiogenesis [37,38]. Since sperm cells do not have ER, the localization of proteins usually located in this structure, such as DERL1, DERL2, and HSP90B1, should be clarified in mature spermatozoa, particularly because their functions and subcellular localization might be different than in somatic cells. 

Mitochondria seems to play a central role in signal transmission to the nucleus under stress conditions [39]. Surprisingly, the amount of proteins hypothetically located in the mitochondria was expected to be higher, since it is one of the remaining organelles in mature spermatozoa, located at the spermatozoa midpiece [40]. However, the identification of few mitochondrial proteins may be justified by the absence of human proteins annotated in the UPR^mt^ biological process. 

Most of the proteins here identified were expressed in most tissues, with only 5% being testis-enriched/specific, highlighting the crucial role of this mechanism for all human cells. The reduced number of UPR proteins associated with infertility phenotypes, and the few phenotypes associated with changes in spermatozoa due to post-testicular causes, reflect the lack of knowledge regarding the role of those proteins in mature spermatozoa, reinforcing the importance of research in this area. Ultimately, the emergence of new evidences for the role of the activation of the UPR signaling pathways under stress conditions in male reproduction and fertility make these processes attractive targets for therapy. Such knowledge may be of great value for the pathophysiological understanding and effective treatment/prevention of male infertility associated to UPR activation.

In the protein–protein interaction (PPI) network, we revealed that the molecular chaperone HSPA5 (BiP) presented the higher degree of connectivity, being highly connected, and was annotated in all four biological processes studied (HSR, IRE1-, ATF6-, and PERK-mediated UPR). This is not surprising since HSPA5 is an ER chaperone usually associated to the luminal domain of unfolded proteins’ sensors (PERK, IRE1, and ATF6), keeping them in an inactive state [5,41]. Under stress conditions, HSPA5 is sequestrated by unfolded proteins activating the ER stress transducers, activating the UPR^ER^ [3]. This in silico analysis revealed that several proteins involved in the stress response are present in mature human sperm; however, no information concerning their activation and functionality in human spermatozoa under stress conditions was available. 

To validate the presence of UPR-related proteins in human ejaculated sperm, we performed immunoblotting and confirmed that the molecular chaperones HSP90, HSP27, HSP60, and eIF2α are present in human spermatozoa. We also showed, for the first time, the presence of HSF1, GADD34, and phosphorylated eIF2α in human spermatozoa. HSF1 is a transcription factor involved in HSR, being, under normal conditions, linked to molecular chaperones such as HSPA1 and HSP90 [42]. In response to several stressors that lead to defects in protein folding, HSF1 dissociates from chaperones, is phosphorylated, and forms a trimer that is translocated to the nucleus, mediating the transcription of heat shock genes [34]. Despite the increased levels of HSF1 mRNA that were observed in the sperm of men with varicocele and with oligozoospermia, the presence of this protein in human ejaculated spermatozoa was never reported. On the other hand, eIF2α is a key player in the stress response, particularly involved in the blockage of protein translation in UPR^ER^ and UPR^mt^. Indeed, the phosphorylation of eIF2α at Ser51 results in inferior levels of translation initiation, thus decreasing the load of synthesized proteins [7,43]. In turn, GADD34 forms a complex with PPP1 to dephosphorylate eIF2α, which fails to prevent protein synthesis [6]. Amaral et al. previously demonstrated that eIF2α was present in human sperm [14]; however, no information regarding its activation in this type of cell is available. Additionally, no information regarding the presence of GADD34 in human sperm also exists. Despite the existence of PPP1CC2 and GADD34 in human spermatozoa, as we showed, we failed to detect the interaction between those proteins using a co-immunoprecipitation approach. However, when interpreting this result, we should be cautious since (i) GADD34 may interact with one of the other isoforms of PPP1 that exist in spermatozoa [26,44], or (ii) the interaction may not be detected due to limitations of the methodology used. Additionally, the fact that spermatozoa are a highly compartmentalized cell type may explain the lack of GADD34–PPP1CC2 interaction. Despite the localization of PPP1CC2 being previously described in human spermatozoa (in the flagellum, midpiece, and the equatorial and post acrosomal regions of the head) [45,46], nothing is known concerning the subcellular location of GADD34. Thus, future immunolocalization studies should be performed to identify the GADD34 location and to determine if the interaction PPP1CC2–GADD34 does not occur, because the two proteins are in distinct compartments of spermatozoa. 

As already mentioned, H_2_O_2_ is the most damaging ROS to human spermatozoa [28]. To investigate whether the UPR is functionally active in human spermatozoa, oxidative stress induction was performed using different concentrations of H_2_O_2_. It was previously shown that low concentration of H_2_O_2_ (10–100 µM) increases the percentage of progressive motile spermatozoa by almost 20% [29]. However, other studies showed that exposure of spermatozoa to 30 and 60 μM of H_2_O_2_ had no effect on sperm motility; at 120 μM of H_2_O_2_, a significant reduction in motility was observed after 120 min; at 600 μM of H_2_O_2_, all the sperm were found immotile within 15 min of incubation and, at much higher concentrations (1200–1500 μM), the effect was immediate [28]. Here we showed that incubation with 120 µM H_2_O_2_ had no significant effect on sperm motility or on the percentage of viable spermatozoa after 2 h incubation, contrary to that observed by Chaki et al. [28]. The exposure of human ejaculated spermatozoa to 600 µM H_2_O_2_ for 15 min and 1200 µM of H_2_O_2_ immediately resulted in a significant decrease of viability and progressive motile spermatozoa, similarly to what was previously described [29]. 

Regarding the activation of stress response pathways, we showed that the levels of proteins associated with the HSR, particularly HSF1, HSP90, and HSP27, significantly increase after induction of oxidative stress, consistent with what happens in somatic cells, including in the testis [47]. Indeed, in response to cellular stress, such as high temperatures, oxidants, and heavy metals, HSF1 dissociates from the chaperones, is phosphorylated, and forms homotrimers that translocate to the promoters of the heat shock genes [42]. It activates the transcription of HSP genes [42], such as HSP70, HSP90, and small HSPs such as HSP27, to assist protein refolding [33], as observed in the present study. Evidences from boar revealed that HSP90 was fundamental in situations of heat stress, being important in the maintenance of spermatozoa motility and mitochondrial membrane potential [48]. Additionally, evidences from humans revealed that spermatozoa of normozoospermic men exposed to frequent scrotal hyperthermia reveal higher levels of HSF1 and HSP70 compared with the control group [49], supporting our hypothesis that HSR may be active in human spermatozoa. 

UPR^mt^ is by far the most unexplored UPR mechanism in mammals, originally triggered by stress in the mitochondrial matrix or in the mitochondrial intermembrane space. The accumulation of mis- or un-folded proteins activate distinctive signal transduction pathways that culminate, as UPR^ER^, in the transcription of heat shock genes or in blockage of protein translation. The mitochondrial chaperone HSP60 is responsible for the direct folding of incoming proteins in the mitochondrial matrix, together with HSP10 [32]. Here we showed that the levels of HSP60 increased after exposure to H_2_O_2_, regardless of the concentration tested, which is consistent to what was previously described in somatic cells. The total levels of eIF2α also increased after oxidative stress induction; however, due to methodological limitations, we did not quantify the levels of p-eIF2. Thus, further studies confirming if this mechanism of the stress response is activated through other branches that do not implicate the blockage of protein synthesis should be done. 

Due to the consistency of the results with what occurs in somatic cells, the belief that transcription and translation do not occur in sperm cells is; thus, called into question or another explanation should be found for the increase in protein levels observed after cellular stress. Despite the general acceptance that nuclear-encoded protein translation does not occur in mature sperm cells due to chromatin packaging by protamines, and that spermatozoa are translationally silent, Yael Gur and Haim Breitbart [50] showed, for the first time, the incorporation of labeled amino acids into polypeptides during sperm capacitation. This study also showed that the inhibition of protein translation has a significant impact on sperm motility, capacitation, and in vitro fertilization rate, as well as on the levels of human and bovine proteins (PKA, PKC, EGFR, progesterone receptor, AKAP110, JAK1, etc.), suggesting that translation may have an important role during these processes [50]. These results were recently confirmed by the work of Zhu et al. [51], which revealed that sperm incubation with mitochondrial translation inhibitor (D-chloramphenicol) suppressed mitochondrial protein synthesis and activity, consequently reducing the linear motility speed of spermatozoa. Thus, if protein translation occurs in mammalian spermatozoa prior to fertilization, it is possible to hypothesize that new proteins are also synthesized in spermatozoa in response to cellular stress, that results in protein misfolding, to correct their folding and ultimately prevent cell damage. Several studies reported the presence of the mRNAs of the UPR main players in human spermatozoa, such as HSP90, HSPA2, HSF1, and others [52,53,54]. To confirm the possibility of transcription, further studies should investigate the levels of these transcripts in human spermatozoa following oxidative stress. Additionally, studies to evaluate if the observed increasing in protein levels can be prevented using translation inhibitors should clarify if these proteins are really being translated under stress conditions.

It is important to be aware that the half-life of the reactive species used, H_2_O_2_, is low compared to the reactive aldehydes and hydrocarbons, generally produced by elevated oxidative stress [55]. These reactive intermediates are usually more lethal and stable in cells. Thus, further studies to confirm these finding need to be performed using other reactive species, such as superoxide anion, and substances, such as paraquat, bleomycin, or ionomycin, that induce oxidative stress in several types of cells. 

To summarize, human spermatozoa have the molecular machinery necessary for UPR activation. The enrichment in molecular chaperones and transcription factors, particularly those involved in HSR and UPR^mt^, indicate that these processes are functional in human spermatozoa. However, future research work should be conducted to clarify the role of UPR signaling pathways activation in male reproduction and fertilization, particularly focusing on the identification of potential therapeutic targets.

## 4. Materials and Methods 

### 4.1. Identification of Proteins Involved in Response to Unfolded Proteins in Human Sperm

An exhaustive literature search was conducted using the PubMed database to identify human sperm proteomic studies published in English (or at least with the abstract in English). A list of all the sperm proteins identified in the proteomic studies available online until 30 April 2018 (studies shown in Table 1) was compiled (Table S1: Human Sperm Proteome). All studies included were performed using ejaculated human spermatozoa (human epididymal spermatozoa and animal models were not included). Only proteomic studies in which a false discovery rate (FDR) < 5% of protein identification was set and proteins identified with at least two peptides were included. To avoid redundancy, all proteins were annotated using the UniProtKB/Swiss-Prot accession number, even when these were not indicated in the original article.

To retrieve human proteins already associated with UPR and HSR, a search was carried out on a Gene Ontology database—AmiGO2 version 2.5.12 (last file loaded on 3 April 2018) (The Gene Ontology Consortium). The term “unfolded protein response” was searched in AmiGO2 query and the GO: 0006986 (“response to unfolded protein”) was selected since it was the most general biological process term related to the unfolded protein response. The lists concerning “response to heat” (GO: 0009498), “IRE1-mediated unfolded protein response”, “PERK-mediated unfolded protein response”, and “ATF6-mediated unfolded protein response” were also retrieved to identify the proteins involved in each biological process. The protein list was restricted to Homo sapiens. The list was downloaded on 22 May 2018 and the output was revised to exclude duplicates. To avoid redundancy, all proteins were annotated using the UniProtKB/Swiss-Prot accession number. To obtain the list of proteins involved in UPR present in human sperm, Venn diagram analysis was performed using the Jvenn tool [56].

### 4.2. Bioinformatic Analysis: Gene Annotation and Involvement of UPR Proteins in Male Infertility-Related Phenotypes

The UniProt database was used to collect information regarding biological process and molecular function and the Human Protein Atlas (HPA) database (available from www.proteinatlas.org) was used to retrieve information regarding tissue expression and subcellular location (data was downloaded on 4 June 2018). To investigate the involvement of the UPR proteins described in human sperm in male fertility, the databases OMIM (genemap.txt file downloaded on 24 May 2018) and DisGeNET (curated gene-disease associations list downloaded on 28 May 2018) were explored. Proteins associated with defects in male fertility were also obtained from the Jackson Laboratories mouse knockout database—MGI (data downloaded on 28 May 2018). The HIPPIE database was used for retrieving human protein–protein interaction (PPI) data (downloaded on 23 March 2019) [57]. This database is regularly updated by incorporating interaction data from major expert-curated experimental PPI databases (such as Bell09, BioGRID, HPRD, IntAct, and MINT). Network analyses were performed using Cytoscape (version 3.6.0) (Institute for Systems Biology and International Consortium of Open Source Developers, Seattle, Washington, USA) [58]. 

### 4.3. Ethical Approval

The study was approved by the Ethics and Internal Review Board of the Hospital Infante D. Pedro E.P.E. (Aveiro, Portugal) (Process number:36/AO; Approved on 14 April 2015) and was conducted in accordance with the ethical standards of the Declaration of Helsinki. All donors signed an informed consent allowing the use of the samples for scientific purposes.

### 4.4. Sperm Preparation

Ejaculated semen samples were obtained from healthy donors by masturbation into a sterile container. Basic semen analyses were performed by qualified technicians according to the World Health Organization (WHO)’s guidelines [59]. After semen liquefaction, sperm cells were washed three times in phosphate-buffered saline (PBS) by centrifugation (500× *g* for 5 min at 23 °C) and resuspended in Sperm Medium Preparation (Origio, Malov, Denmark) until the final concentration desired. 

### 4.5. Whole Cell Lysates

Sperm cells were lysed in 1× RIPA buffer (0.5 M Tris-HCl, pH 7.4, 1.5 M NaCl, 2.5% deoxycholic acid, 10% NP-40, 10 mM EDTA) (Millipore Iberica S.A.U., Madrid, Spain) supplemented with protease inhibitor (1 mM PMSF) for 30 min on ice, and centrifuged at 16,000× *g* for 15 min at 4 °C. The supernatant was used for the subsequent steps (sperm soluble extract). Protein concentration was measured using the bicinchoninic acid (BCA) assay (Pierce Biotechnology, Waltham, Massachusetts, USA) following the manufacturer’s instructions and the final absorbance was measured at 562 nm in a microplate reader (TECAN, Genius, Männedorf, Switzerland).

### 4.6. Oxidative Stress Induction

To determine the effects of oxidative stress on human spermatozoa, sperm cells were incubated with H_2_O_2_ at final concentrations of 120, 600, and 1200 µM for 2 h, 15 min, and 30–60 sec, respectively. The concentrations and times of H_2_O_2_ were selected based on previous studies showing the effects of different concentrations of H_2_O_2_ on sperm function, particularly in motility and viability, over time [28,29]. Ten million of cells per condition were used, and sperm cells maintained for 2 h at 37 °C in were used as negative control. Each condition was tested on samples from five individual donors.

### 4.7. Cell Viability Assays

Human spermatozoa viability was measured using the CellTiter 96^®^ AQueous Non-Radioactive Cell Proliferation Assay (Promega, Madison, Wisconsin, USA) according to the manufacturer’s guidelines. Briefly, 5 × 10^6^ spermatozoa/100 µL medium were added to 20 µL of CellTiter 96^®^ and incubated for 1 h at 37 °C. Absorbance was measured at 490 nm using the Infinite^®^ 200 PRO (TECAN, Genius, Männedorf, Switzerland). The reduction of tetrazolium compounds has previously been used as a reliable and rigorous assessment of spermatozoa viability.

### 4.8. Motility Assays

The influence of H_2_O_2_ exposure on sperm motility parameters was assessed using the Sperm Class Analyzer CASA System (Microptic S L, Barcelona, Spain) with SCA^®^ v6.2 software (Microptic S L, Barcelona, Spain). Samples and controls (2 µL) were loaded into individual chambers of Leja Standrat Count 4 chamber slide 10 µm depth (Leja Products B.V., Nieuw-Vennep, The Netherlands) which were pre-heated at 37 °C. This temperature was maintained while at least 1000 sperm cells/measurement were evaluated. 

### 4.9. Antibodies

The anti-PPP1CC2 antibody was raised in rabbit against the specific PPP1CC2 C-terminal peptide (VGSGLNPSIQKASNYRNNTVLY) and has been described previously [60]. Antibody against normal rabbit IgG (sc-2027), used as negative control for immunoprecipitation and against HSF1 (sc-177757), GADD34 (sc-373815), p-HSP27 (Ser 82) (sc-166693), and HSP27 (sc-13132), used for Western blot analysis, was obtained from Santa Cruz Biotechnology (Dallas, Texas, USA). Antibodies against eIF2α (9722S) and p-eIF2α (Ser 51) (119A11) were obtained from Cell Signaling Technology Europe Inc (Leiden, The Netherlands). The anti-HSP90 (13171-1-AP) and anti-HSP60 (15282-1-AP) were obtained from Proteintech (Manchester, United Kingdom). The infrared IRDye^®^680RD anti-rabbit (926-68071) and IRDye^®^800CW anti-mouse (926-32210) secondary antibodies were obtained from LI-COR Biosciences (Lincoln, Nebraska, USA).

### 4.10. Western Blot 

To investigate the presence of HSF1, GADD34, eIF2α, and p-eIF2α (Ser51) in human sperm, sperm extracts corresponding to 30 µg of protein were resolved by 10% SDS-polyacrylamide gel electrophoresis (PAGE), and proteins were electro-transferred onto nitrocellulose membranes (Amersham, 0.45 µm). To evaluate the levels of HSP90, HSP60, HSF1, HSP27, p-HSP27, eIF2α, and p-eIF2α, 5 × 10^6^ spermatozoa lysed in 30 µL of 1× RIPA buffer (0.5 M Tris-HCl, pH 7.4, 1.5 M NaCl, 2.5% deoxycholic acid, 10% NP-40, 10 mM EDTA) (Millipore Iberica S.A.U., Madrid, Spain) supplemented with protease inhibitor (1 mM PMSF) were loaded per condition and resolved by 10% SDS-polyacrylamide gel electrophoresis (PAGE), and proteins were electro-transferred into nitrocellulose membranes (Amersham, 0.45 µm). Non-specific protein-binding sites on the membrane were blocked with 5% bovine serum albumin (BSA) or 5% non-fat milk in Tris-buffered saline containing 0.1% Tween 20 (TBST) for 1 h at room temperature. The blots were then incubated with primary antibodies for 1 h at RT (PPP1CC2, HSP60, HSP90, and HSF1) or overnight at 4 °C (GADD34, HSP27, p-HSP27, eIF2α, and p-eIF2α). After incubation, the blots were washed three times for 10 min each with TBST and incubated with the appropriate secondary antibody for 1 h at room temperature. Blots were immunodetected using the Odyssey Infrared Imaging System (LI-COR^®^ Biosciences, US) and the results were analyzed in the Image Studio Lite Version 5.2.5 Software (LI-COR Biosciences, Lincoln, Nebraska, USA).

### 4.11. Co-Immunoprecipitation of PPP1CC2

For the detection of PPP1CC2/GADD34 interaction, 30 million human sperm cells per condition were lysed in 1000 μL of 1× RIPA buffer (0.5 M Tris-HCl, pH 7.4, 1.5 M NaCl, 2.5% deoxycholic acid, 10% NP-40, 10 mM EDTA) (Millipore Iberica S.A.U., Madrid, Spain) supplemented with protease inhibitor (1 mM PMSF) for 30 min on ice, and centrifuged at 16,000× *g* for 15 min at 4 °C. Ninety percent of the supernatant was used for the subsequent steps (sperm soluble fraction). Ten percent of the soluble fraction was saved and the pellet (insoluble fraction) was re-suspended in 1000 µL 1% sodium dodecilsulfate (SDS) (sperm insoluble fraction).

RIPA supernatant extracts were pre-cleared using 50 µL of Dynabeads^®^ Protein G (Db) (Life Technologies AS., Madrid, Spain) per condition, for 1 h at 4 °C with rotation. The crosslinking of the antibody (Ab) to the beads were performed by adding 3 µL of homemade anti-PPP1CC2 antibody or anti-IgG antibody to 50 µL of Dynabeads protein G per condition. The mixture was allowed to incubate for 20 min at room temperature with rotation. The Db–Ab complex was washed twice with conjugation buffer, re-suspended in 5 mM bis(sulfosuccinimidyl)suberate (Bs3) solution and incubated for 30 min with rotation. Then, to quench the crosslinking reaction, 12.5 µL of quenching buffer was added, incubated for 15 min with rotation at room temperature, and, finally, the sample was washed twice with PBS 1×. A direct co-immunoprecipitation approach was performed by incubating the pre-cleared soluble fraction with the Db–Ab complex for 2 h at room temperature with rotation. After incubation, the supernatant was removed to a new microtubule and stored (unbound IP fraction) and the beads were washed two times with 500 µL PBS for 15 min at 4 °C with rotation. After washing, the beads were re-suspended in 1× loading buffer and boiled for 5 min. The eluted fraction was then collected (IP fraction).

### 4.12. Statistical Analysis

To investigate the impact of H_2_O_2_ exposure in human sperm motility, vitality, and the levels of UPR-related proteins, we conducted Mann–Whitney U tests. The assumptions of the statistical techniques used were validated. The statistical analysis was performed using Stata 13 for Windows (StataCorp, College Station, Texas, USA).

## 5. Conclusions

In this study we showed that UPR-related proteins are present in human spermatozoa, including those involved in UPR^ER^. We also showed, for the first time, the presence of (i) HSF1, a transcription factor involved in the recognition of unfolded proteins and transcription of HSP genes; (ii) GADD34, a PPP1 regulator involved in the control of eIF2α phosphorylation state; and (ii) phosphorylated eIF2α (Ser51), the active state of eIF2α, which results in the blockage of translation. We confirmed that high concentrations of H_2_O_2_ significantly decreased human sperm motility and viability, and showed that the levels of UPR-related proteins, such as HSF1 and HSPs, significantly increase. Together, these observations gave us a first insight about the presence and activation of UPR in human spermatozoa. 

## Figures and Tables

**Table 1:** Human sperm proteomic studies. The number of proteins collected in each study and the number of UPR proteins identified are also indicated.

References	Total Proteins Identified in Each Study	Total Proteins Identified without Duplicates in All Studies	UPR Proteins Identified in Each Study	Total UPR Proteins Identified in Human Sperm Proteome
Sharma et al. 2013 [13]	72	7622	6	97
Amaral et al. 2014 * [14]	6198		93	
Amaral et al. 2014 [15]	1624		33	
McReynolds et al. 2014 [16]	49		3	
Y. Liu et al. 2014 [17]	1974		37	
F. Liu et al. 2015 [18]	22		1	
Jumeau et al. 2015 [19]	94		0	
Wang et al. 2016 * [20]	2132		42	
Bogle et al. 2016 [21]	264		8	
Vandenbrouk et al. 2016 [22]	235		0	
Garin-Muga et al. 2016 [23]	111		0	
Intasqui et al. 2017 [24]	572		22	

* Reviews including several proteomic studies of human spermatozoa.

## Supplementary Materials

Supplementary Materials can be found at https://www.mdpi.com/1422-0067/20/21/5518/s1. Table S1—Human sperm proteome. List of 7622 different proteins along with their literature references. Table S2—UPR-related proteins. List of 178 proteins linked to “response to unfolded protein” without duplicates. Table S3—Sperm UPR-related proteins. List of 97 proteins annotated in “unfolded protein response” and present in human sperm, along with information concerning molecular function, biological process, subcellular location, tissue expression, and infertility-related phenotypes. Table S4—UPR-specific processes. List of proteins associated with “response to heat shock”, “PERK-mediated UPR”, “IRE1-mediated UPR”, and “ATF6-mediated UPR” present in human sperm. Table S5—Effect of hydrogen peroxide on sperm motility, viability, and on the levels of UPR-related proteins—statistical analysis.

## Abbreviations

UPR	Unfolded protein response
H_2_O_2_	Hydrogen peroxide
HSF1	Heat shock factor 1
GADD34	DNA damage-inducible protein GADD34
HSP	Heat shock protein
eIF2α	Eukaryotic translation initiation factor-2
ER	Endoplasmic reticulum
HSR	Heat shock response
UPR^ER^	Endoplasmic reticulum unfolded protein response
UPR^mt^	Mitochondrial unfolded protein response
HPA	Human Protein Atlas
PPP1	Serine/threonine-protein phosphatase 1
ROS	Reactive oxygen species
PPP1CC2	Phosphoprotein phosphatase 1 subunit gamma 2
SD	Standard deviation
PPI	Protein–protein interaction
