# Supplementary material for: First Insights on the Presence of the Unfolded Protein Response in Human Spermatozoa
Source: Int J Mol Sci. 2019 Nov 5;20(21):5518. doi: 10.3390/ijms20215518 (PMC6861958; doi:10.3390/ijms20215518)
Supplement: Supplementary file 1 [file ijms-20-05518-s001.zip › Supplementary files/Supplementary File 3 - Original Images Figure 4.pdf]

# Figure 4

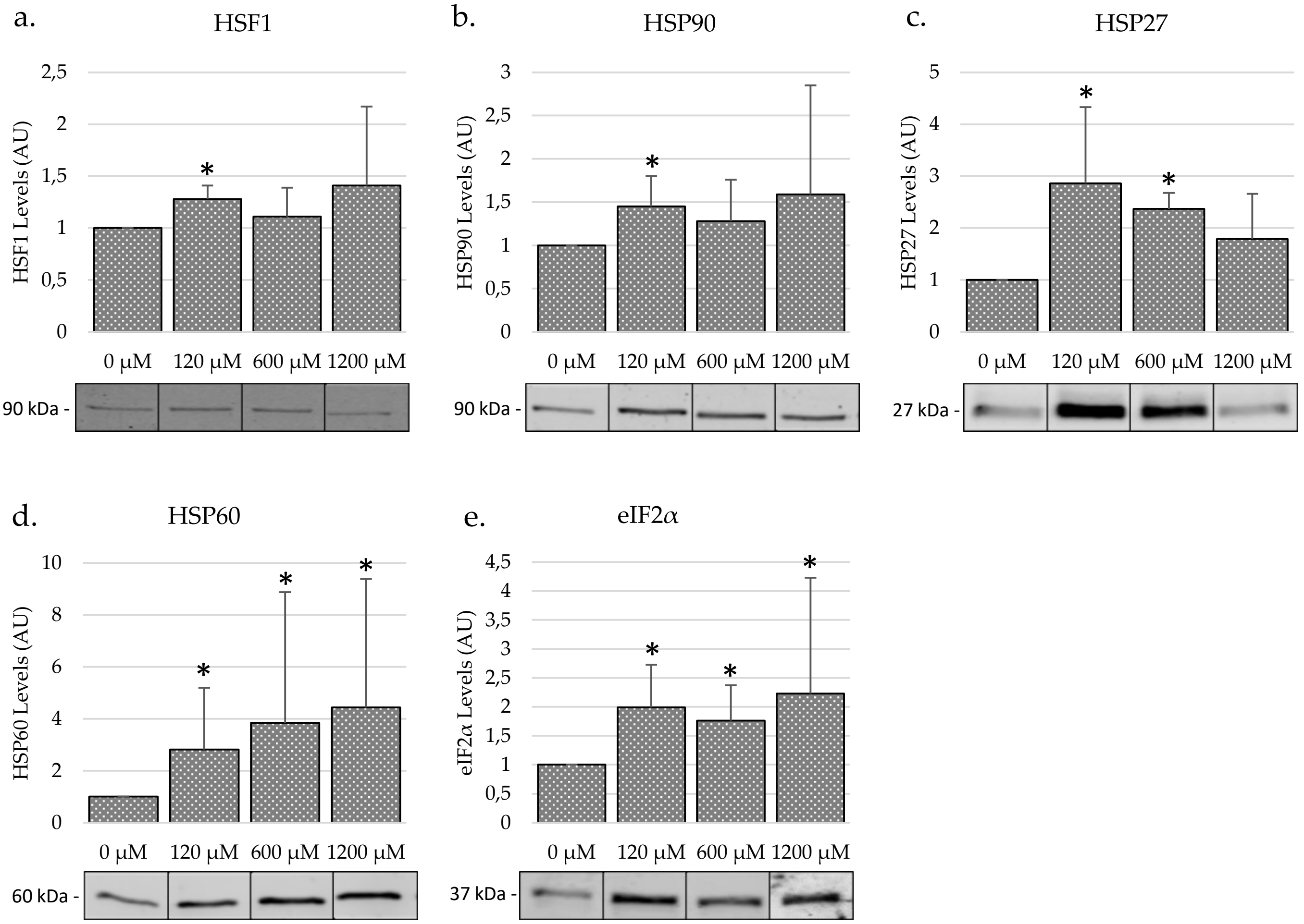

a.
HSF1
HSF1 Levels (AU)
2,5
2
1,5
1
0,5
0
*
0 µM
120 µM
600 µM
1200 µM
90 kDa -
b.
HSP90
HSP90 Levels (AU)
3
2,5
2
1,5
1
0,5
0
*
0 µM
120 µM
600 µM
1200 µM
90 kDa -
c.
HSP27
HSP27 Levels (AU)
5
4
3
2
1
0
*
*
0 µM
120 µM
600 µM
1200 µM
27 kDa -
d.
HSP60
HSP60 Levels (AU)
10
8
6
4
2
0
*
*
*
0 µM
120 µM
600 µM
1200 µM
60 kDa -
e.
eIF2α
eIF2α Levels (AU)
4,5
4
3,5
3
2,5
2
1,5
1
0,5
0
*
*
*
0 µM
120 µM
600 µM
1200 µM
37 kDa -

# Figure 4 – Original Images

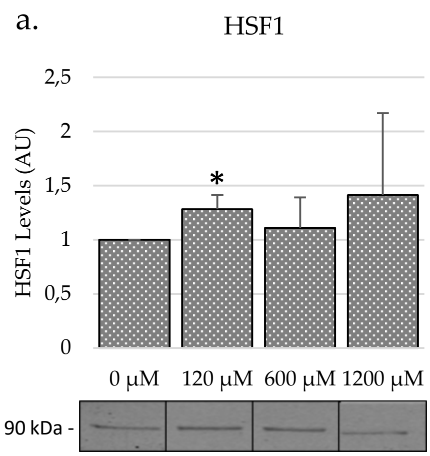


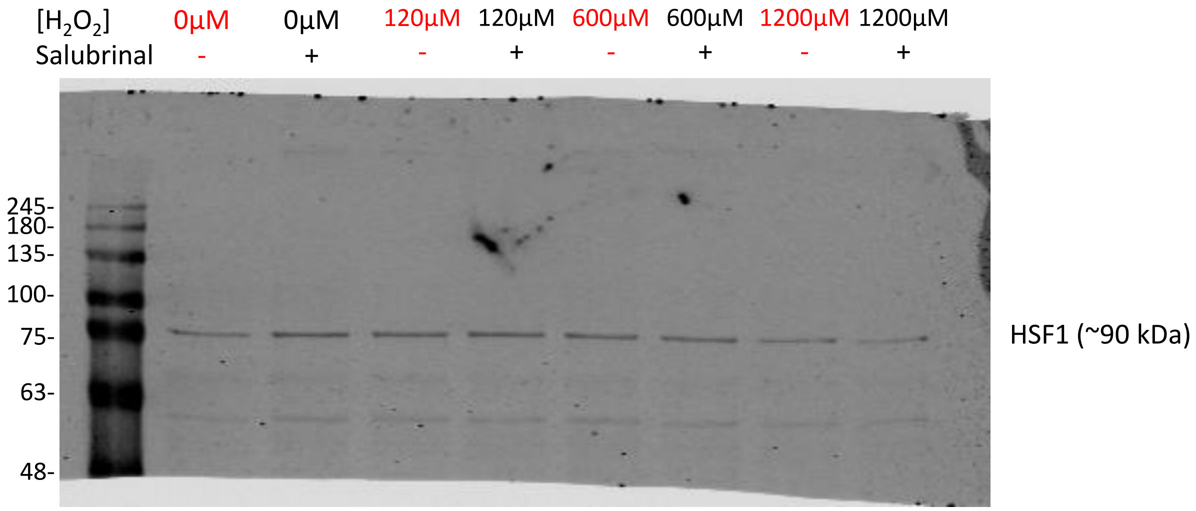


Note: Only the results of red conditions are presented and discussed in the paper.

# Figure 4 – Original Images

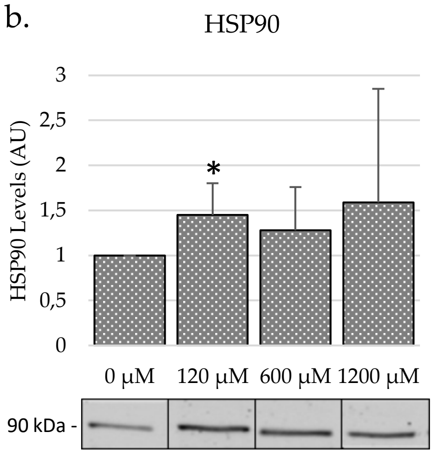


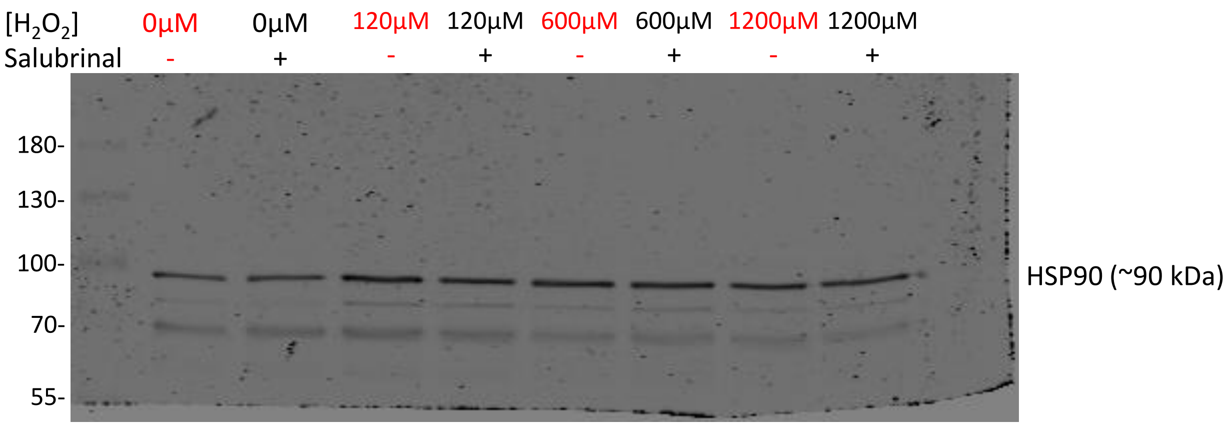


Note: Only the results of red conditions are presented and discussed in the paper.

# Figure 4 – Original Images

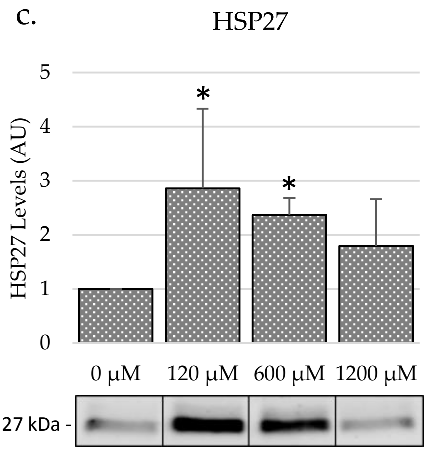


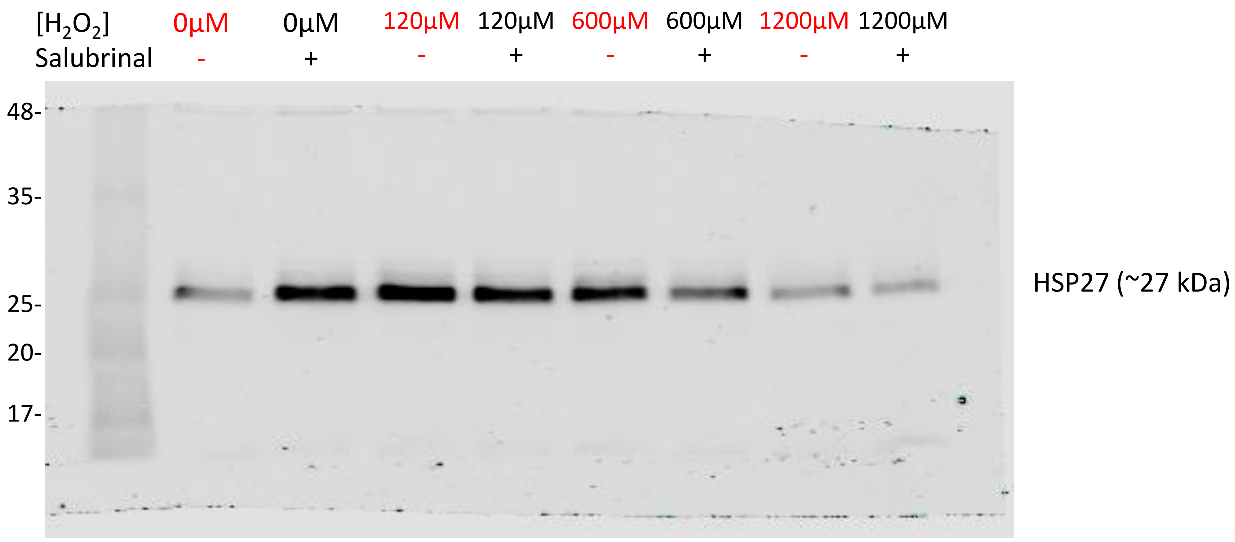


Note: Only the results of red conditions are presented and discussed in the paper.

# Figure 4 – Original Images

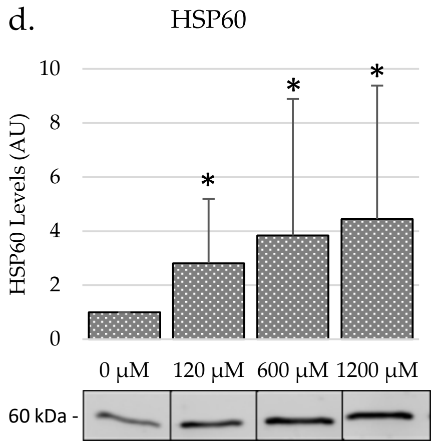


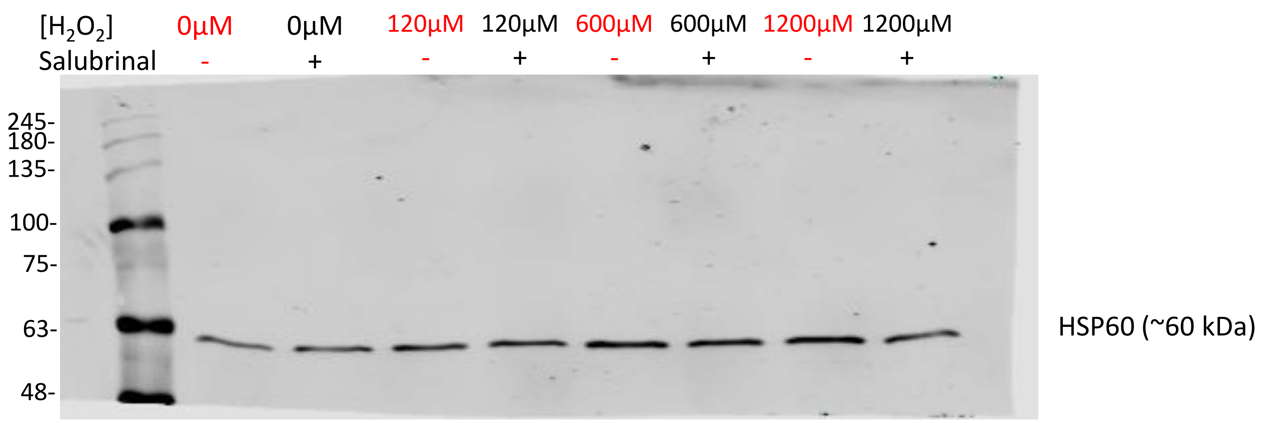


Note: Only the results of red conditions are presented and discussed in the paper.

# Figure 4 – Original Images

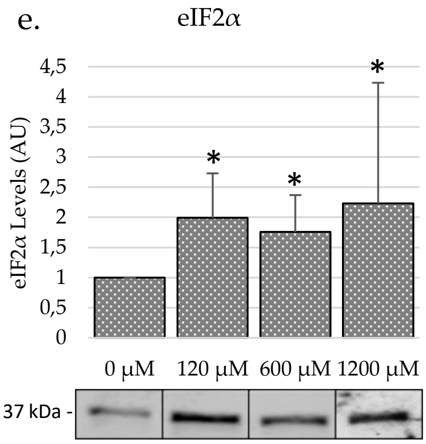


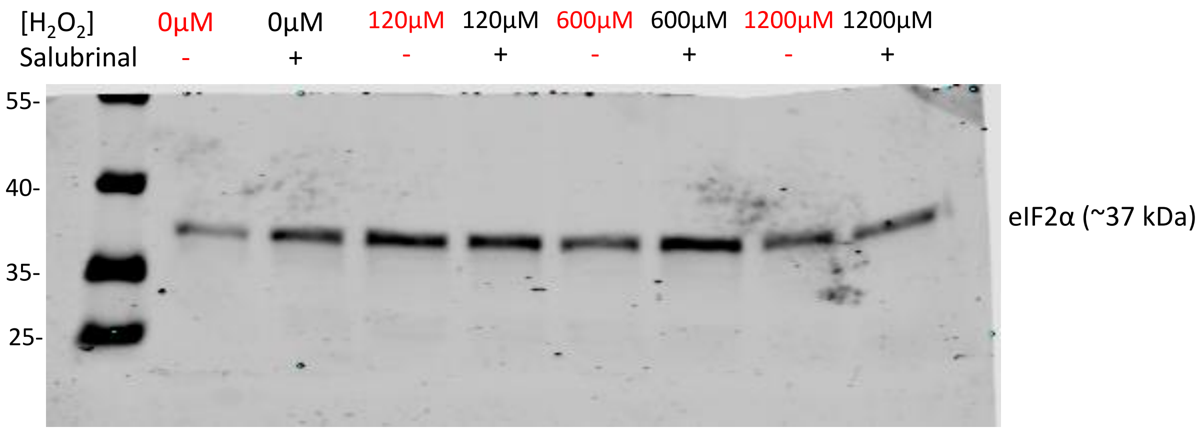


Note: Only the results of red conditions are presented and discussed in the paper.
