# Supplementary material for: First Insights on the Presence of the Unfolded Protein Response in Human Spermatozoa
Source: Int J Mol Sci. 2019 Nov 5;20(21):5518. doi: 10.3390/ijms20215518 (PMC6861958; doi:10.3390/ijms20215518)

Figure 2

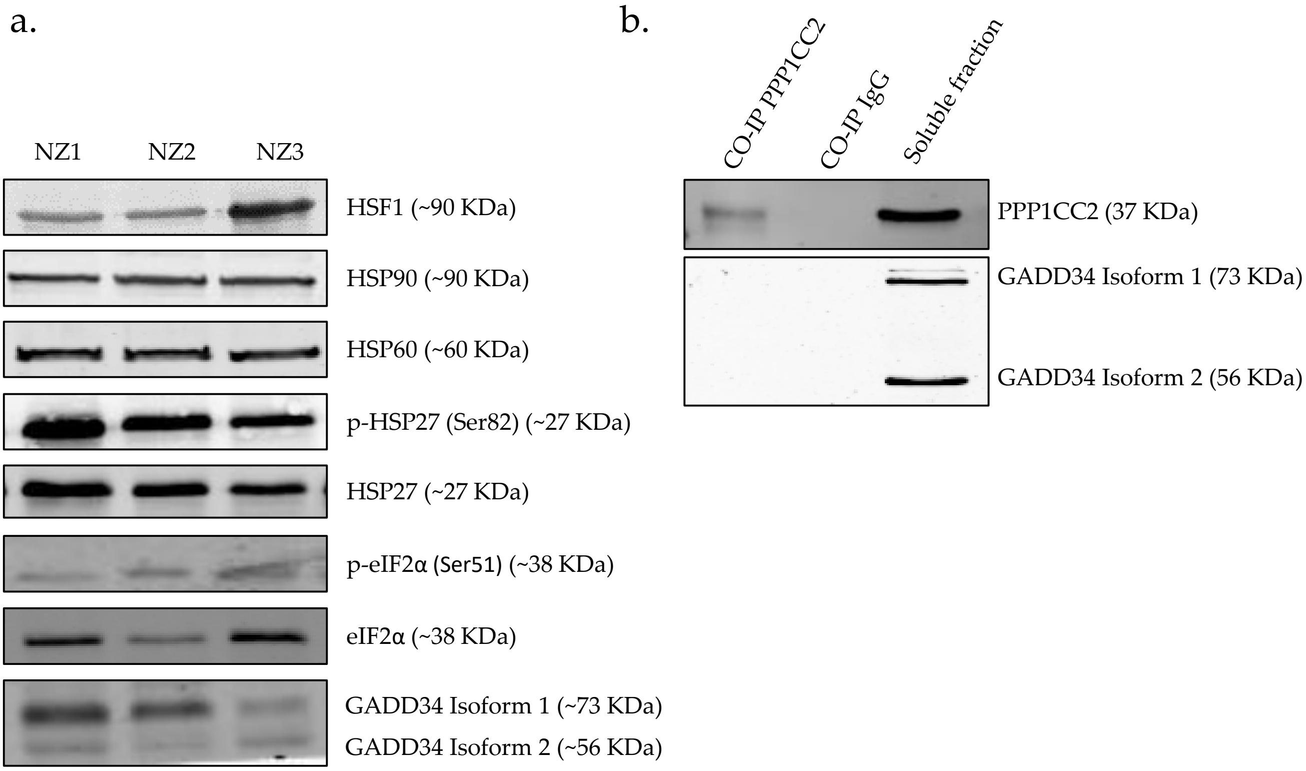
a.
NZ1
NZ2
NZ3
HSF1 (~90 KDa)
HSP90 (~90 KDa)
HSP60 (~60 KDa)
p-HSP27 (Ser82) (~27 KDa)
HSP27 (~27 KDa)
p-eIF2α (Ser51) (~38 KDa)
eIF2α (~38 KDa)
GADD34 Isoform 1 (~73 KDa)
GADD34 Isoform 2 (~56 KDa)
b.
CO-IP PPP1CC2
CO-IP IgG
Soluble fraction
PPP1CC2 (37 KDa)
GADD34 Isoform 1 (73 KDa)
GADD34 Isoform 2 (56 KDa)

Figure 2.a – Original Images

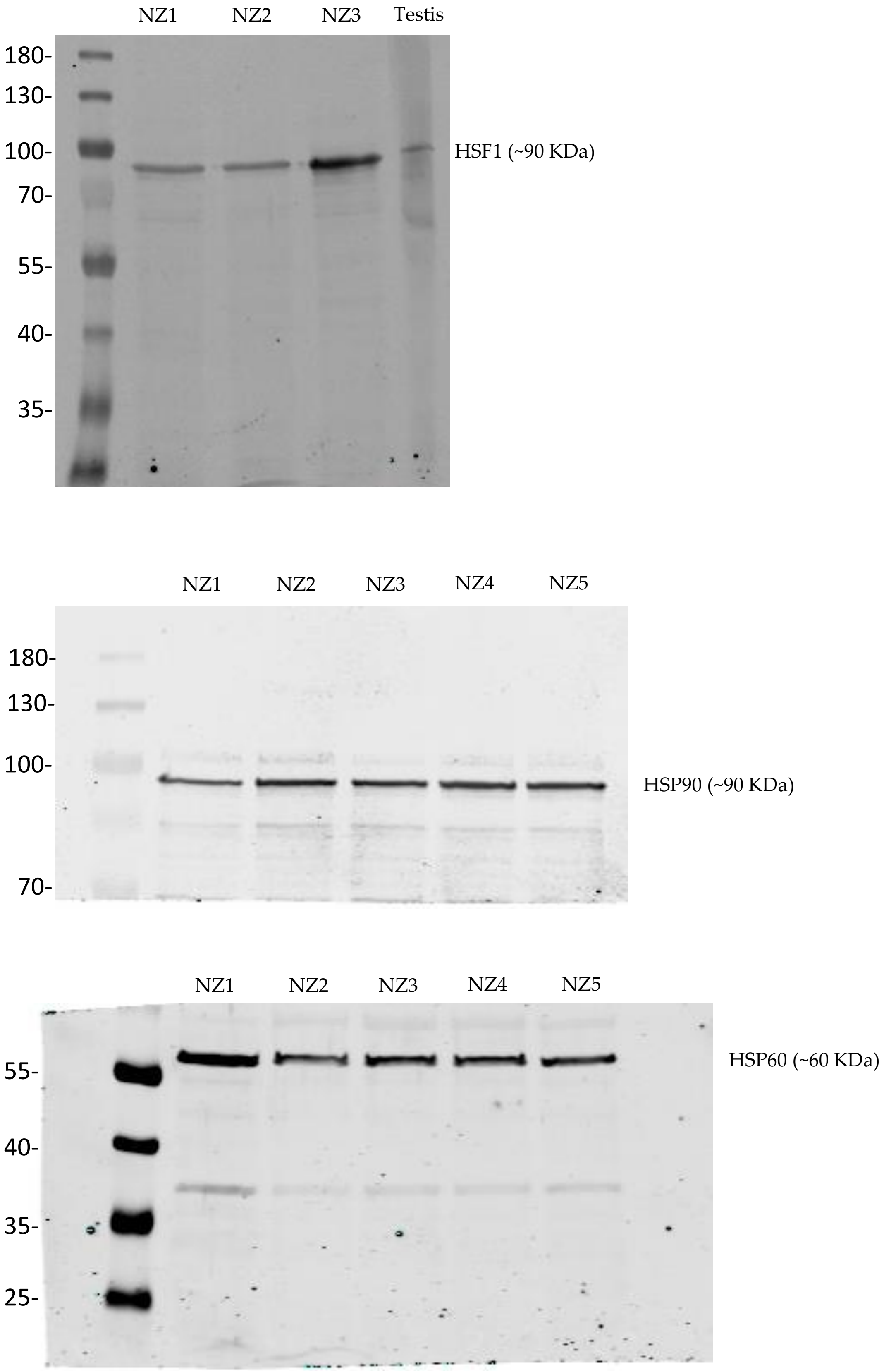

Figure 2.a – Original Images

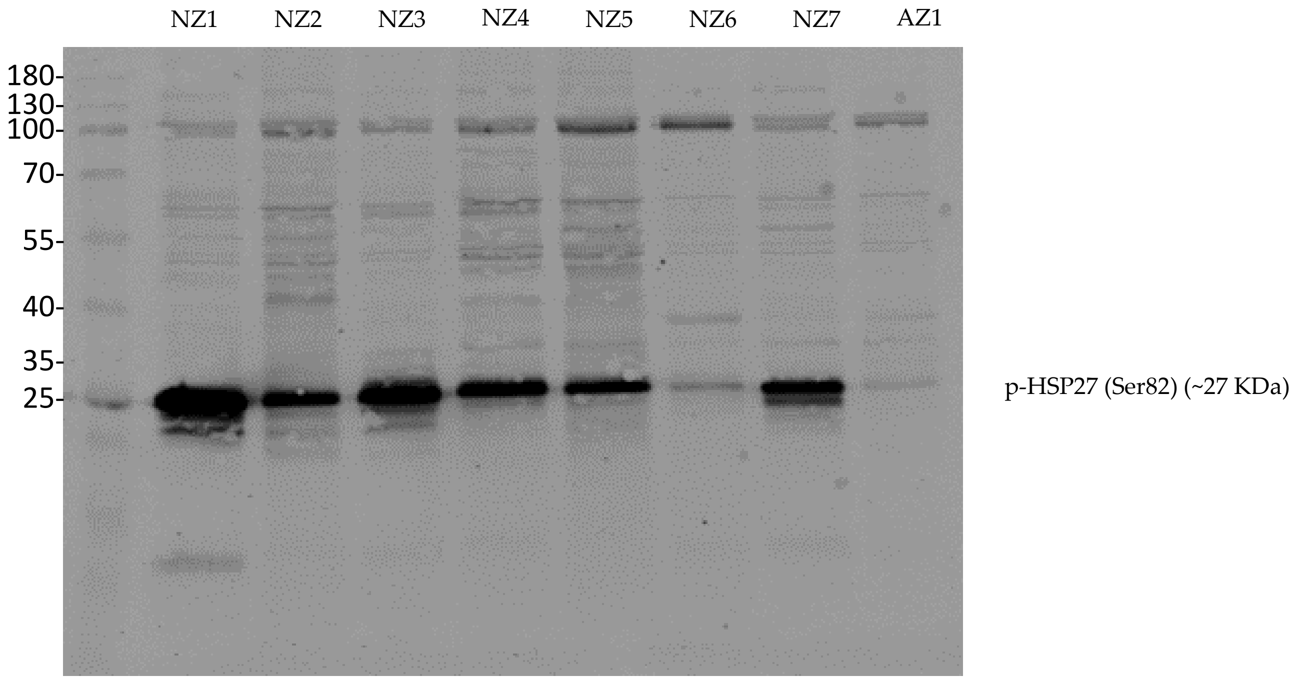

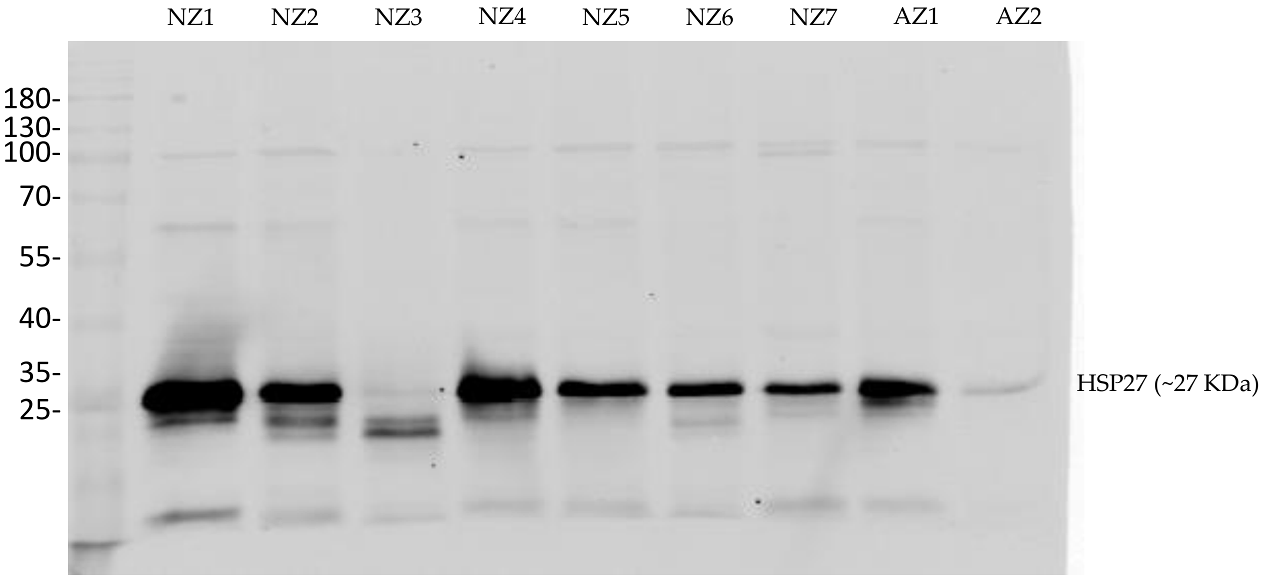

Figure 2.a – Original Images

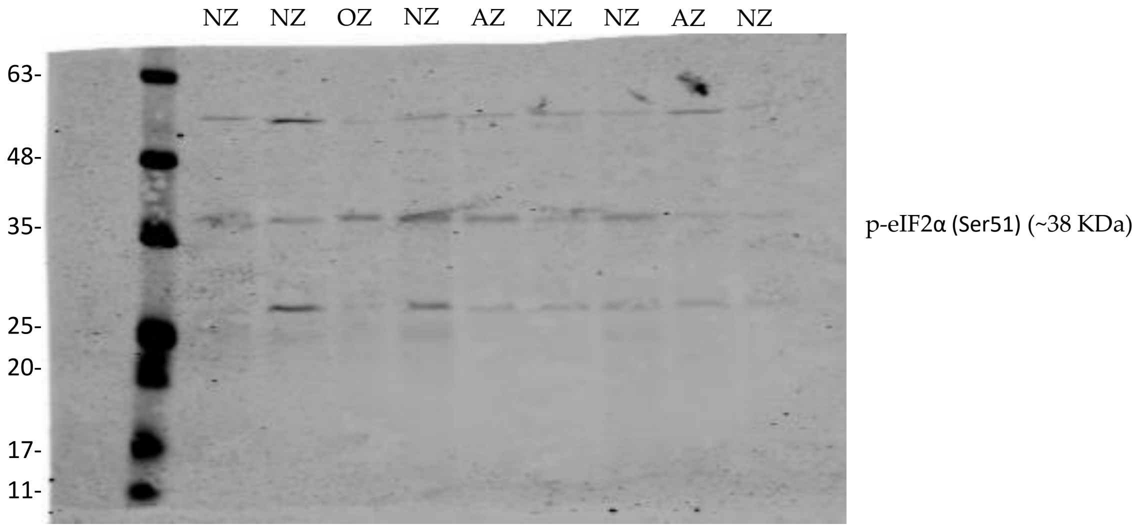

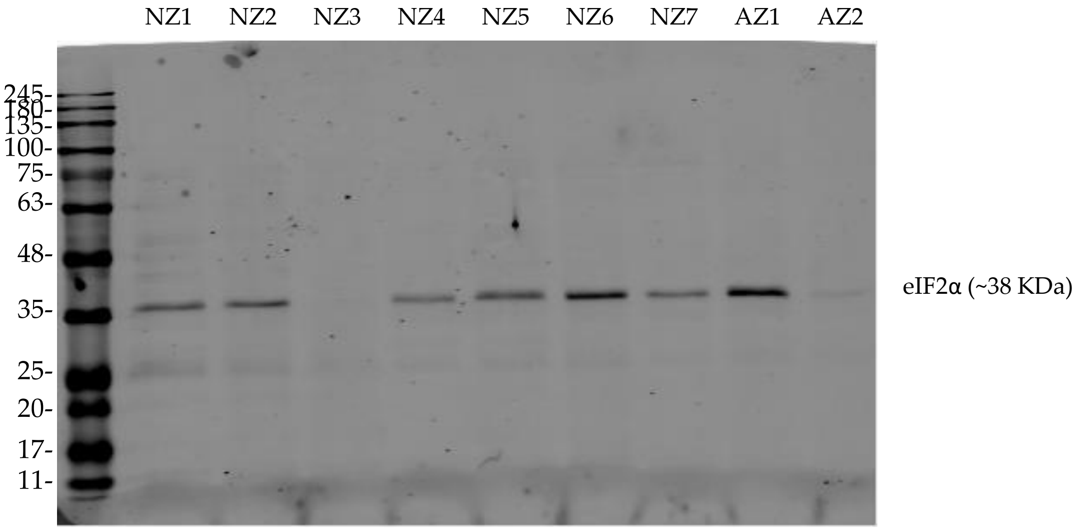

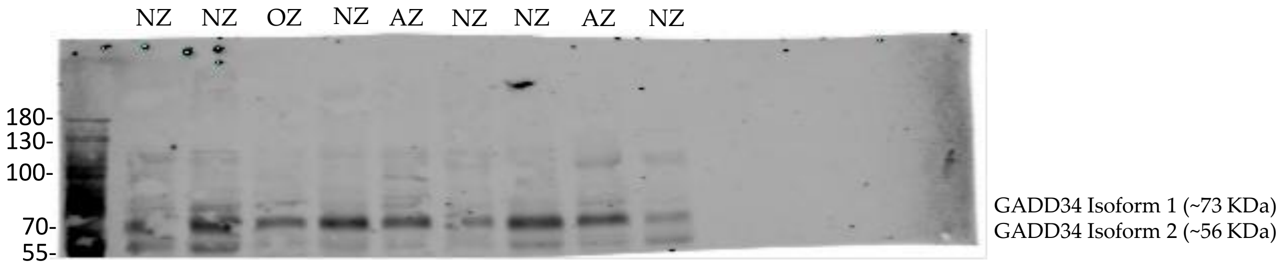

Figure 2.b – Original Images

b.

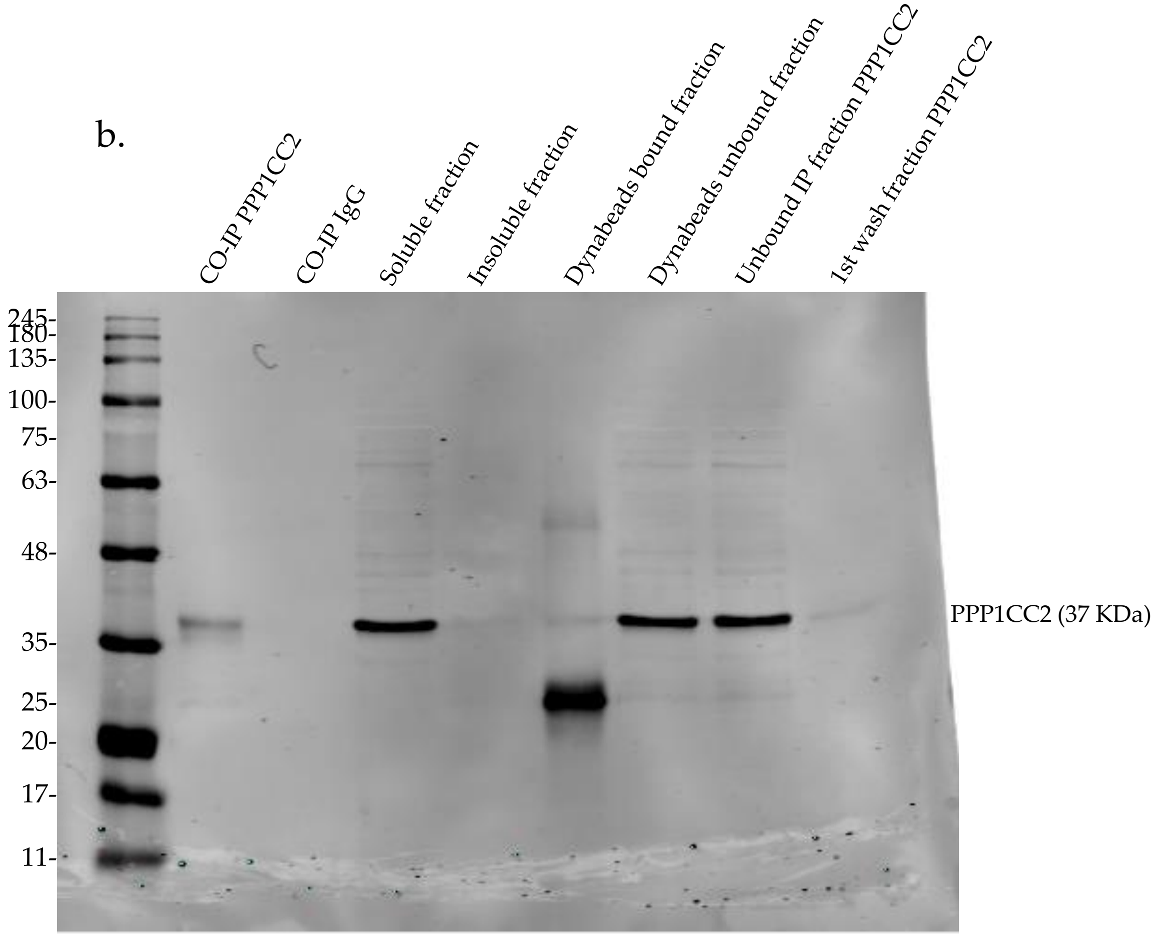

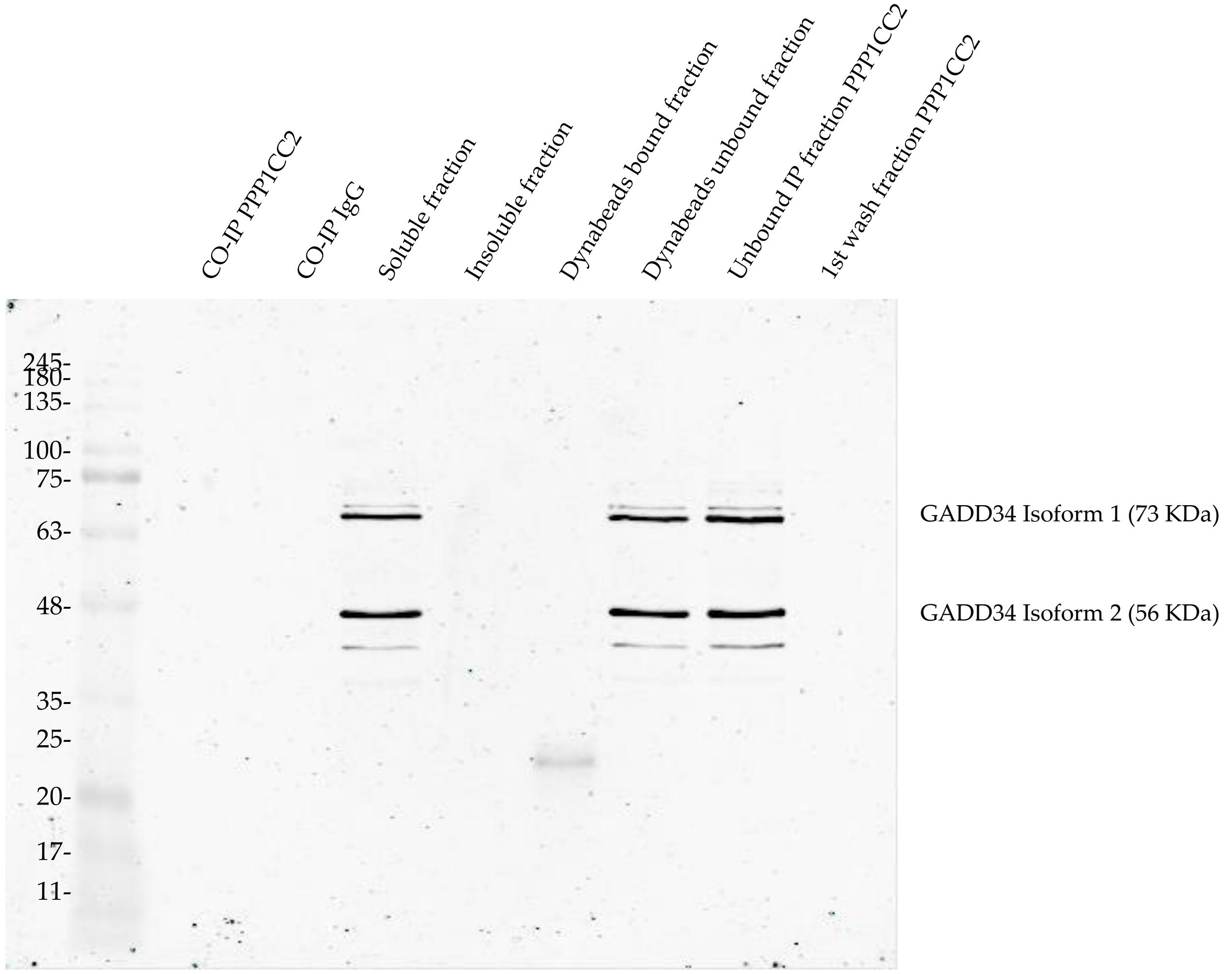

Supplement: Supplementary file 1 [file ijms-20-05518-s001.zip › Supplementary files/Supplemetary File 2 - Original Images Figure 2.pdf]
