# Supplementary figures and images for: First Insights on the Presence of the Unfolded Protein Response in Human Spermatozoa
Source: Int J Mol Sci. 2019 Nov 5;20(21):5518. doi: 10.3390/ijms20215518 (PMC6861958; doi:10.3390/ijms20215518)

Figure 2 – Positive control (**testis**)

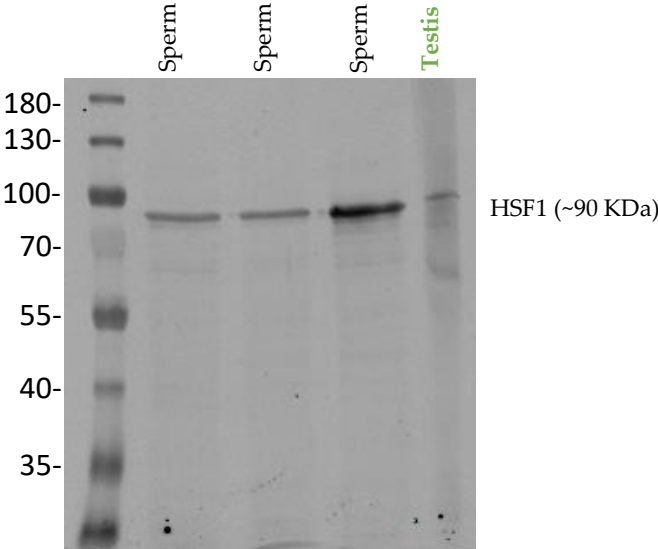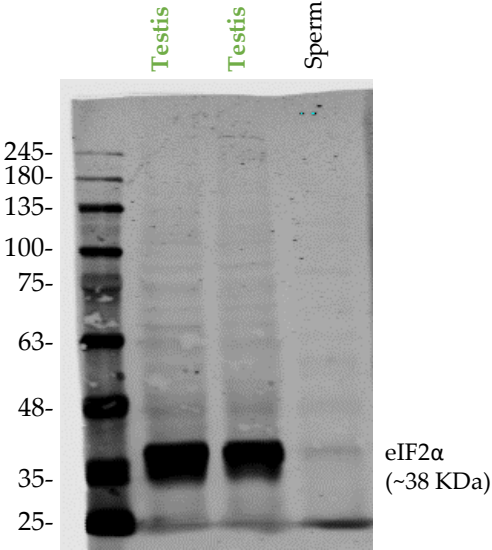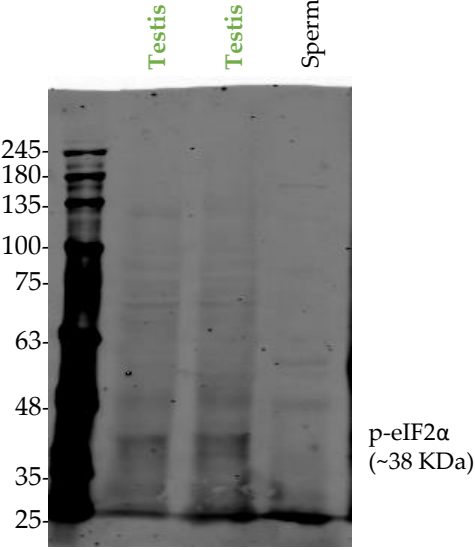

Supplement: Supplementary file 1 [file ijms-20-05518-s001.zip › Supplementary files/Supplementary File 1 - Positive controls used in immunoblotting.pdf]
